# Accelerometer-derived sleep metrics in mild and difficult-to-treat asthma

**DOI:** 10.1186/s13223-024-00874-y

**Published:** 2024-01-14

**Authors:** Varun Sharma, Helen Clare Ricketts, Femke Steffensen, Anna Goodfellow, Duncan S Buchan, Douglas C Cowan

**Affiliations:** 1 Institute of Infection, Immunity and Inflammation, College of Medical, Veterinary & Life Sciences, University of Glasgow, Glasgow, UK; 2 Glasgow Royal Infirmary, Clinical Research Facility, Glasgow, UK; 3 Division of Sport and Exercise, School of Health and Life Sciences, University of the West of Scotland, Glasgow, UK; 4 Respiratory Department, Glasgow Royal Infirmary, 84 Castle Street, Walton Building, Fourth Floor, Glasgow, G4 0SF UK

**Keywords:** Accelerometers, Asthma, Difficult asthma, Severe asthma, Sleep medicine

## Abstract

**Introduction:**

Poor sleep health is associated with increased asthma morbidity and mortality. Accelerometers have been validated to assess sleep parameters though studies using this method in patients with asthma are sparse and none have compared mild to difficult-to-treat asthma populations.

**Methods:**

We performed a retrospective analysis from two recent in-house trials comparing sleep metrics between patients with mild and difficult-to-treat asthma. Participants wore accelerometers for 24-hours/day for seven days.

**Results:**

Of 124 participants (44 mild, 80 difficult-to-treat), no between-group differences were observed in sleep-window, sleep-time, sleep efficiency or wake time. Sleep-onset time was ~ 40 min later in the difficult-to-treat group (*p* = 0.019).

**Discussion:**

Broadly, we observed no difference in accelerometer-derived sleep-metrics between mild and difficult-to-treat asthma. This is the largest analysis of accelerometer-derived sleep parameters in asthma and the first comparing groups by asthma severity. Sleep-onset initiation may be delayed in difficult-to-treat asthma but a dedicated study is needed to confirm.

## Introduction

Uncontrolled asthma can affect sleep quality as increased nocturnal symptoms are synonymous with uncontrolled disease. However, short or excessive sleep duration and poor sleep quality are risk factors for asthma exacerbations and healthcare usage, poorer quality of life and mortality [1]. Accelerometers provide a novel opportunity to evaluate sleep parameters relative to asthma severity.

Accelerometery has been validated against polysomnography for measurement of sleep-related variables in asthma and sleep measures can be obtained using a validated algorithm for wrist-worn accelerometers without the use of accompanying sleep diaries [2]. These tri-axial devices measure acceleration, allowing estimates of physical activity, sedentary time and sleep. Accelerometers are less cumbersome than sleep diaries, encouraging adherence, provide additional data such as sleep onset and efficiency, and are a cost-effective option compared to polysomnography.

We hypothesised that sleep patterns differ between mild and difficult-to-treat asthma populations. We performed a cross-sectional, proof-of-concept analysis comparing sleep parameters from participants with mild and difficult-to-treat asthma utilising accelerometer technology.

## Materials and methods

Data for this analysis was retrieved from two recent local trials approved by the West of Scotland Regional Ethics Committee (references 16/WS/0200 and 18/WS/0216) and undertaken between 2017 and 2021: one of pulmonary rehabilitation in difficult-to-treat asthma associated with raised body mass index (BMI) alongside a sub-study of activity levels in mild asthma, and a second trial studying weight loss in difficult-to-treat asthma and obesity (trial identifiers: NCT03630432, NCT03858608). Full trial protocols are described elsewhere [3, 4]. Both trials were funded by an NHS Greater Glasgow and Clyde Endowment Fund, and none of the contributors to the fund had any input in trial design, results or interpretation, nor any input into this retrospective analysis. All participants provided written consent for data use in future studies.

Briefly, difficult-to-treat asthma was defined as per SIGN/BTS and GINA guidelines [5, 6], including presence of characteristic symptoms, reversibility (≥ 12% and 200mls increase in FEV_1_ post-bronchodilator) or bronchial hyper-reactivity on bronchial challenge testing; asthma treatment with high-dose inhaled corticosteroid (ICS); poor asthma control (Asthma Control Questionnaire score > 1.5) or ≥ 2 exacerbations requiring oral corticosteroids (OCS) or ≥ 1 asthma exacerbation requiring hospitalisation in the preceding 12 months. Patients with mild active asthma (asthma treatment within the preceding 12 months) were recruited from primary care. Mild disease was categorised by maximum preventer treatment with moderate-dose ICS/long-acting β-agonist combination, ACQ ≤ 1.5, < 2 exacerbations requiring OCS treatment and no hospital admissions with asthma in the preceding 12 months.

As part of the trial assessments, participants wore an ActiGraph wGT3X-BT accelerometer (ActiGraph, Pensacola, USA) on their non-dominant wrist continually for 7 days (excluding bathing). Devices were initialised to capture data at 30 Hz. Raw data was downloaded using ActiLife software (v.6.14.3; ActiGraph) and saved as .gt3x files and converted to .csv files. Data was exported into R v4.1.2 (R Foundation for Statistical Computing, Vienna, Austria) for subsequent processing using the GGIR package (v2.6.0).

Among the variables extracted were number of nights devices were worn; mean sleep window time (time from initial sleep-onset to waking); mean sleep time (accumulated sustained inactivity sojourns overnight); sleep efficiency (sleep time: sleep window); sleep-onset time and wake time. Time variables were described as hours and minutes or 24-hour clock where appropriate. Variables were non-parametric and so summarised as median (IQR) and compared between mild and difficult-to-treat asthma groups using the Mann-Whitney U test. Data was analysed using IBM SPSS Statistics (version 28.0) and significance was set at 0.05.

## Results

Of 133-patient data-sets available, nine were excluded due to lack of data (defined ≤ 3 nights use), leaving 124 participants (44 with mild asthma, 80 with difficult-to-treat asthma). Of the 124, 56% were female, median (IQR) age was 57 (47, 64) years and the majority were never and ex-smokers (56% and 38% respectively). Baseline characteristics (Table 1) showed differences between mild and difficult-to-treat participants in atopy, weight, BMI, asthma control and quality of life, long-acting β-agonist (LABA) use and number of annual exacerbations.Higher baseline fractional exhaled nitric oxide (FeNO) and peripheral eosinophils were observed in the difficult-to-treat asthma group compared to mild asthma.

Table 2 summarises sleep-metric findings. Overall, the median number of nights accelerometery was available was 6 (6, 6). Median sleep time was 6hrs35mins (5hrs2mins, 7hrs45mins), with a median sleep window time of 7hrs49 mins (6hrs29mins, 8hrs56mins) and median sleep efficiency of 85% (81, 90). Median time of sleep-onset was 00:08 (23:02, 01:23) and wake time 07:54 (06:48, 09:22).

No differences were observed in sleep time, sleep window, sleep efficiency or wake time between the mild and difficult-to-treat groups, though sleep-onset time was later in the difficult-to-treat asthma group (00:24; 23:16, 02:02) compared to mild asthma (23:41; 22:52, 00:45; *p* = 0.019). In the overall dataset (I.e., mild and difficult-to-treat groups together), Spearman’s rank showed no correlation between sleep-onset time and ACQ (marker of asthma control); rho = 0.049, *p* = 0.589. Additionally, both unadjusted and adjusted (correcting for weight) linear regression using sleep-onset time as the dependent variable and ACQ as the independent variable showed no relationship between asthma control and sleep-onset time: unadjusted F(1,122) = 0.28, *p* = 0.866; adjusted for weight F(2,121) = 0.160, *p* = 0.852.

## Discussion

We observed no differences in sleep duration or efficiency between mild and difficult-to-treat groups, but whilst there was no difference in wake time, there was a later time of sleep-onset in the difficult-to-treat group which may reflect greater difficulty in sleep initiation in this cohort. The clinical significance of this difference (~ 40 min) is uncertain, however, interestingly correlation and regression analysis suggest this difference is not related to asthma control even when adjusted for weight, a key factor in sleep health. There was a significant between-group difference in proportion of participants with regular LABA use and it is feasible that β-agonist-mediated stimulation could be related to the delay in sleep initiation in the difficult-to-treat group. Compared to the recommended sleep duration, patients from our cohort appear to be on the lower side (6.59 h; 5.04, 7.75) suggesting poorer sleep health despite good sleep efficiency. Factors associated with delayed sleep initiation and reduced sleep duration in difficult-to-treat asthma therefore remain to be elucidated and require further study.

Our results are similar to a study performed in 56 healthy adults (mean age 24.5 ± 4.5 years) also using ActiGraph devices (non-dominant wrist) without sleep logs that showed (mean ± SD) sleep time (6 h 56 min ± 49 min), sleep window (7 h 59 min ± 51 min) and sleep efficiency (87%±4), as well as similar sleep-onset (00:05 ± 90 min) and wake times (08:20 ± 84 min) [7]. A small study of 10 patients with mild-to-moderate asthma [2] showed reduced sleep time of 5 h 54 min ± 74 min with a similar mean sleep window time of 7 h 34 min ± 40 min. However, this study is clearly limited by the small sample size.

Our retrospective analysis has potential limitations. Firstly, groups were not equally weighted with more patients with difficult-to-treat asthma than mild asthma. Secondly, the initial trials data did not include objective assessments of daytime or nocturnal sleep (e.g., Epworth sleep score, Pittsburgh sleep quality index), nor any sleep logs. Thirdly, this analysis was not powered to assess sleep outcomes. Finally, this analysis did not account for factors such as sleep-disordered breathing that may influence outcomes, which should be addressed in future studies. Despite this, key strengths of our study are the sample size, higher than in previous studies, and observed excellent tolerance of accelerometer use (93%). To our knowledge this is the first comparison of mild and difficult-to-treat asthma sleep outcomes using accelerometery and we highlight a difference in sleep initiation between groups unrelated to asthma control and weight. Further study is warranted to explore the relationship between asthma severity and sleep-metrics and whether interventions targeting sleep health can improve asthma outcomes.

In summary, patients with difficult-to-treat asthma may have delayed initiation of sleep compared to mild asthma, though this observation appears to be independent of asthma control and obesity. Other sleep parameters are broadly comparable to the general population. Accelerometers are well tolerated, offer more pragmatism than polysomnography and can be used to assess sleep outcomes in asthma but dedicated trials are needed before any definitive conclusions can be drawn.

**Table 1:** Baseline characteristics

Variable	Overall *n* = 124	Mild asthma *n* = 44	Difficult-to-treat *n* = 80	*p* value
Age, years	57 (47 to 64)	60 (48 to 72)	56 (48 to 65)	0.843
Female sex, no. (%)	69 (55.6)	25 (56.8)	44 (55.0)	0.845
Smoking status: Never smoker Ex-smoker Current smoker	69 (55.6) 47 (37.9) 8 (6.5)	30 (68.2) 12 (27.3) 2 (4.5)	39 (48.8) 35 (43.8) 6 (7.5)	0.114
Atopy, no. (%)	55 (44.4)	6 (13.6)	49 (61.3)	**< 0.001**
Weight, kg	84.6 (73.0 to 99.5)	75.3 (65.7 to 84.9)	92.3 (76.8 to 107.8)	**< 0.001**
BMI, kg/m^2^	31.0 (26.5 to 36.4)	25.7 (21.9 to 29.6)	33.6 (28.8 to 38.5)	**< 0.001**
SABA	122 (98.4)	42 (95.5)	80 (100.0)	0.124
LABA/ICS	101 (81.5)	21 (47.7)	80 (100.0)	**< 0.001**
Maintenance prednisolone, no. (%)	28 (22.6)	n/a	28 (35.0)	n/a
Biologic, no. (%)	13 (10.5)	n/a	13 (16.3)	n/a
Prednisolone boosts	2 (0 to 4)	0 (0 to 0)	4 (3 to 6)	**< 0.001**
ACQ6	1.7 (0.5 to 3.0)	0.4 (0.0 to 0.8)	2.7 (1.9 to 3.6)	**< 0.001**
AQLQ overall	4.6 (3.8 to 6.2)	6.4 (5.9 to 6.9)	4.0 (3.3 to 4.8)	**< 0.001**
FeNO, ppb	23 (16 to 45)	21 (16 to 26)	33 (12 to 54)	**0.023**
Eosinophils, x10^9^/L	0.2 (0.1 to 0.4)	0.1 (0.0 to 0.2)	0.3 (0.2 to 0.5)	**0.017**

Continuous variables described as median (interquartile range)

Categorical variables described as n (%)

*p*-value compares mild vs. difficult-to-treat groups with Mann Whitney U for continuous and chi square or Fisher’s exact for categorical variables

Abbreviations: ACQ6 (Asthma Control Questionnaire), AQLQ (Asthma Quality of Life Questionnaire), FeNO (fractional exhaled nitric oxide), LABA/ICS (long-acting β-agonist/inhaled corticosteroid combination inhaler), ppb (parts per billion), SABA (short-acting β-agonist inhaler)

**Table 2:** Sleep parameters of asthma patients overall and by disease severity

Variable	Overall *n* = 124	Mild *n* = 44	Difficult-to-treat *n* = 80	*p* value*
No. of nights used	6 (6, 6)	6 (5, 6)	6 (6, 6)	0.333
Sleep time	6:35 (5:02, 7:45)	6:50 (6:05, 7:45)	6:26 (4:56, 7:44)	0.353
Sleep window	7:49 (6:29, 8:56)	8:03 (7:02, 8:50)	7:38 (6:08, 8:59)	0.339
Sleep efficiency (%)	85.4 (81.0, 90.2)	86.3 (82.1, 90.5)	85.4 (80.2, 90.0)	0.471
Sleep onset	00:08 (23:02, 01:23)	23:41 (22:52, 00:45)	00:24 (23:16, 02:02)	**0.019**
Wake onset	07:54 (06:48, 09:22)	07:41 (06:43, 08:13)	08:03 (06:48, 10:01)	0.097

Variables described as median (IQR) in hours:mins unless specified.

*Mann Whitney U test comparing mild vs. difficult-to-treat asthma groups

## Data Availability

Data is available upon reasonable request.
